# Use of rapid diagnostic techniques in ICU patients with infections

**DOI:** 10.1186/s12879-014-0593-1

**Published:** 2014-11-28

**Authors:** Almudena Burillo, Emilio Bouza

**Affiliations:** Clinical Microbiology and Infectious Diseases Department, Hospital General Universitario Gregorio Marañón, Doctor Esquerdo 46, Madrid, 28007 Spain; Instituto de Investigación Sanitaria Gregorio Marañón, Madrid, Spain; Medicine Department, School of Medicine, Universidad Complutense de Madrid, Madrid, Spain; CIBER de Enfermedades Respiratorias (CIBERES CB06/06/0058), Madrid, Spain

**Keywords:** Rapid diagnosis, Clinical laboratory techniques, Intensive care unit, Microbiology

## Abstract

**Background:**

Infection is a common complication seen in ICU patients. Given the correlation between infection and mortality in these patients, a rapid etiological diagnosis and the determination of antimicrobial resistance markers are of paramount importance, especially in view of today's globally spread of multi drug resistance microorganisms. This paper reviews some of the rapid diagnostic techniques available for ICU patients with infections.

**Methods:**

A narrative review of recent peer-reviewed literature (published between 1995 and 2014) was performed using as the search terms: Intensive care medicine, Microbiological techniques, Clinical laboratory techniques, Diagnosis, and Rapid diagnosis, with no language restrictions.

**Results:**

The most developed microbiology fields for a rapid diagnosis of infection in critically ill patients are those related to the diagnosis of bloodstream infection, pneumonia -both ventilator associated and non-ventilator associated-, urinary tract infection, skin and soft tissue infections, viral infections and tuberculosis.

**Conclusions:**

New developments in the field of microbiology have served to shorten turnaround times and optimize the treatment of many types of infection. Although there are still some unresolved limitations of the use of molecular techniques for a rapid diagnosis of infection in the ICU patient, this approach holds much promise for the future.

## Review

### Background

Although intensive care units (ICUs) have fewer than ten percent of the total number of beds in most hospitals, more than 20 percent of all nosocomial infections are acquired in ICUs and carry substantial morbidity, mortality, and expense [1]-[4]. The most common clinically significant infections observed in the ICU are intravascular catheter-related bloodstream infection (CR-BSI), ventilator associated pneumonia (VAP), and catheter associated urinary tract infection (CA-UTI).

In addition, multidrug-resistant (MDR) pathogens are evermore frequently isolated in ICUs [5],[6] and this hinders the initiation of appropriate, effective antibiotic therapy, which correlates with excess mortality [7]-[9].

In this setting, a rapid etiologic microbiological diagnosis is mandatory. This paper reviews some of the rapid diagnostic techniques available for ICU patients with infections.

## Main text

### Rapid diagnosis of catheter-related bloodstream infections (CR-BSI)

A diagnosis of CR-BSI should be based on microbiological identification of the catheter as the source of bloodstream infection, and may be performed with or without catheter removal [10].

Attempts to establish the role of the catheter in episodes of BSI are justified by the following: a high proportion of the suspicious of CR-BSI are note confirmed after catheter removal and culture [11], and many CR-BSI can be managed empirically without immediately removing the catheter [12]-[14]. Central venous catheter (CVC) removal limits vascular access, and diagnostic methods exist that do not require catheter removal [15].

### Rapid procedures that do not require catheter removal

The conservative approach to CR-BSI diagnosis (i.e., without catheter withdrawal) is highly convenient. Conservative procedures include differential paired quantitative blood cultures (comparison of colony counts in peripheral vein blood versus catheter hubs), superficial cultures (semiquantitative cultures of skin around the portal of entry and of catheter hubs), and a method comparing time to positivity between concurrent blood cultures of peripheral vein and catheter hub samples, named "differential time to positivity" (DTTP) [16]-[18].

#### Paired central/peripheral cultures

A ratio or differential colony count ≥3:1 cfu/mL of bacteria from the catheter-drawn blood cultures compared to percutaneously-drawn blood cultures is usually accepted as a prove of CR-BSI. This cutoff shows a sensitivity (Se) of around 80% and specificity (Sp) of 90-100% [19].

Blood should be drawn from all hubs, representing the different catheter lumens [20]. This technique is usually performed with lysis-centrifugation tubes. Blood is inoculated in tubes containing the cell-lysing agent saponin, followed by vortexing and centrifugation. Then, after removing the supernatant (lysate), the concentrate is plated on agar medium and the plates incubated overnight before counting. The tubes need to be processed within 8 hours of inoculation [21]. Drawbacks of this technique include: the manual and individual processing of each individual sample, the risk of contamination, the risk of exposure of laboratory technicians to blood and the high cost [19].

#### Differential time to positivity (DTTP)

DTTP supporting CRBSI diagnosis is defined as a difference in time to positivity of ≥2 h between a CVC blood culture and a peripheral blood culture, or between 2 CVC blood cultures from different lumens of a multi-lumen catheter [10],[22],[23]. The DTTP test is conducted using a continuous-monitoring automated blood culture system. This method requires inoculating the same amount of blood in each culture bottle. For multiple lumen catheters, blood should be drawn from all ports [20],[24]. To ensure accurate results, the first milliliters of blood drawn from the catheter should be used for culture. Then, bottles must be sent to the laboratory and incubated as soon as they arrive there. Depending on the type of catheter (short- *vs.* long-term) and the patient, the test shows a Se of 86-93%, Sp of 87-92%, positive predictive value (PPV) of 85-88% and negative predictive value (NPV) of 89-95% [22],[24],[25].

DTTP is nowadays the main technique to assess CR-BSI used in most microbiology departments. Caution in interpretation should be applied in patients receiving antimicrobial agents [22]. The validity of DTT, however, has been recently questioned by Kaasch *et al*. [26]., that found a poor diagnostic performance (Se 37%, Sp 77%, PPV 46%, NPV 70%, validity 63%) in patients with CR-BSI caused by *Staphylococcus aureus*. However, they failed to adhere to instructions of utmost importance related to the protocol. The microbiology service was not available on a 24/7 basis, suggesting long pre-incubation periods before introducing the bottles in the automated blood culture machine, possibly leveling times to positivity of paired cultures, thus invalidating the diagnostic procedure [27],[28].

Our group recently demonstrated that the DTTP threshold applied to bacterial CR-BSI is not applicable in cases of CR-BSI caused by *Candida* spp. [29].

#### Superficial cultures (combined exit-site *and*hub cultures)

We call 'superficial cultures' to the combination of semiquantitative cultures independently obtained from the 2 cm of skin surrounding the catheter insertion site and the various hubs.

The threshold for positivity of these semiquantative cultures is 15 cfu per plate.

Growth of <15 cfus per plate of the same microbe from both the insertion site culture and catheter hub/s culture/s strongly suggests that the catheter is not the source of the BSI. Superficial cultures are justified only in cases of suspected CR-BSI (targeted cultures) in which they serve to rule out CR-BSI owing to their high sensitivity and good negative predictive value [16].

Gram staining of skin and hub swabs may also be helpful for the rapid diagnosis of CR-BSI [30].

Recently Bouza *et al*. compared the use of paired blood cultures, superficial cultures and DTTP for the diagnosis of CR-BSI without catheter removal [31]. DTTP showed a better sensitivity and negative predictive capacity than paired blood cultures to detect catheter tip colonization (96.4% and 99.4% *vs.* 71.4% and 95.6%, respectively) (Table 1). However, central/peripheral paired blood cultures showing a ratio >5:1 provided the best specificity (97.7%) for a diagnosis of CR-BSI. The three tests showed a high negative predictive capacity. If a negative result was obtained in any of the three tests, it was possible to rule out catheter colonization and CR-BSI reasonably well.

**Table 1:** **Validity indices (95% confidence interval) for three commonly used methods of detecting catheter-related bloodstream infection**

Measure	Semiquantitative superficial cultures	Differential quantitative blood cultures	Differential time to positivity
Sensitivity	78.6 (59.0-91.7)	71.4 (51.3-86.8)	96.4 (81.7-99.9)
Specificity	92.0 (87.0-95.6)	97.7 (94.3-99.4)	90.3 (85.0-94.3)
Positive predictive value	61.1 (43.5-76.9)	83.3 (62.6-95.3)	61.4 (45.5-75.6)
Negative predictive value	96.4 (92.4-98.7)	95.6 (91.4-98.1)	99.4 (96.6-99.9)
Accuracy	90.2 (85.3-93.9)	94.1 (90.0-96.9)	91.2 (86.4-94.7)

From reference [31].

### Rapid diagnosis of sepsis

The diagnosis of BSI among critically ill patients is a major challenge. Blood cultures are still considered the gold standard diagnostic procedure since pathogens may be isolated and subjected to antibiotic susceptibility testing (AST). In effect, the use of blood cultures in septic shock patients as part of the compliance with six or more interventions of the 6-hour resuscitation bundle of the 'surviving sepsis campaign' has been related to a reduction in mortality [32].

Blood cultures, however, are time-consuming and slow. They only detect viable microorganisms and show a low sensitivity for slow growing, intracellular and fastidious microorganisms. Overall positivity may be as low as 30-40% despite proper implementation of standard procedures, adequate blood volume collection and a high clinical suspicion of BSI.

Molecular techniques are ever-evolving to provide faster and more sensitive results along with the direct identification of responsible pathogens [33]-[36]. These techniques are likely to impact soon clinical decision-making and antibiotic treatment.

Existing commercial nucleic acid testing (NAT) diagnostic tests are all based on a similar procedure: pathogen lysis, nucleic acid extraction and purification, amplification of nucleic acids by PCR, and identification by various methods, such as ELISA-based hybridization, fluorescence-based real-time detection, liquid or solid phase microarray detection, sequencing and database recognition [34]. The reader is referred to Afshari *et al*. [34] for a comprehensive review of the tests commercially available today.

Pathogen-specific assays are even capable of detecting genes encoding resistance to antibiotics, such as *mecA* in staphylococci or *van* genes in enterococci.

A recent meta-analysis on the use of LightCycler SeptiFast revealed a Se and Sp of 80% and 95%, respectively, for this technique to detect bacteremia, and of 61% and 99%, respectively, to detect fungemia [36]. However, the bacteremia outcome subgroup showed high variation. The turnaround time of the technique was 6 hours.

In general terms, there are still important shortcomings of molecular techniques. For instance, the lack of an appropriate gold standard since blood cultures are unable to detect many true cases of infection; emphasis on microbiological rather than clinical assessment; no guidance for targeting appropriate clinical situations; and the potential for wrong interpretation of results if no expert assistance is available [37],[38].

Ideally, tests should provide relevant information 2-6 hours after samples are taken on which to base the choice of treatment. Under real-life conditions, there are often considerable delays due to practical issues, such as availability of staff outside daily routines or batch analysis of samples [39]. Test sensitivity needs to be improved to detect clinically relevant low bacterial loads and fastidious microorganisms. They should be able to distinguish between living and dead bacteria, especially for patients on antibiotics. They should also be able to clarify the impact of DNAemia in cases of clinical signs of BSI. For instance, in a recent paper on the combined use of blood cultures and SeptiFast to predict complicated BSI in cases of staphylococcal or *Candida* infection, the authors found that patients with a positive SeptiFast result between days 3 and 7 after a positive blood culture had an almost 8-fold-higher risk of developing a complicated bloodstream infection [40].

At present, molecular tests are used to complement the results of traditional culture, especially in serious clinical situations such as ICU patients with severe sepsis [37]. They also have the potential to be a cost-effective strategy to manage sepsis [41]. However, conventional blood cultures remain necessary because of the high incidence of multidrug-resistant bacteria in ICU patients and the need for AST to establish adequate treatment.

### Other helpful rapid tests for the diagnosis of sepsis

Matrix assisted laser desorption/ionization time-of-flight (MALDI-TOF) mass spectrometry (MS) serves to identify isolated colonies of bacteria and fungi, and can also be used directly on positive blood culture broths in under one hour after the technician has been alerted of growth. This procedure is now replacing biochemical and gene sequencing methods for organism identification because it is easily implemented, highly accurate, inexpensive and fast [42]-[44]. Some 5 to 10 ml of broth from a single positive blood culture bottle are needed for this technique. However, in most reports to date, identification yields are greater for Gram negative organisms than Gram positives or yeasts. To improve diagnosis, different sample preparation methods for positive blood cultures have been tested. Most of these methods include preincubation with different detergent concentrations (e.g., 5% saponin, 5% sodium dodecyl sulfate 'sDS-, 0.1% Tween 80) or use of the Sepsityper kit (Bruker Daltonik GmbH, Bremen, Germany) [45],[46].

In a recent study conducted at our center, we assessed the use of MALDI-TOF MS as a routine method for the identification of microorganisms directly from positive blood culture bottles (BCB) [47]. The turnaround time for results ranged from 20 to 30 minutes, similar to that reported in other studies. Analysis by bacteremia episode led to the complete identification of 814 out of 1000 episodes (81.4%). As expected, Gram negative microorganisms were better identified than Gram positives or yeasts. However, by comparing spectral peaks we were able to differentiate between *Streptococcus pneumoniae* and *Streptococcus mitis* or *Streptococcus oralis*.

MALDI-TOF MS identification is available for clinicians within hours of a working shift, as opposed to 18 h for a conventional identification method. Moreover, although further improvement of sample preparation for polymicrobial BCBs is required, the identification of more than one pathogen in the same BCB provides a valuable indication of unexpected pathogens when their presence may remain undetected by Gram staining.

It has already proved useful for improving the adequacy of antibiotic treatment of bacteremia [48].

### Diagnostic and prognostic biomarkers in sepsis

More than 180 molecules have been described as potential biological makers of sepsis. These molecules include C-reactive protein (CRP), procalcitonin (PCT), several cytokines, and cell surface markers [49], though only 20% have been assessed for use in the diagnosis of sepsis [50].

C-reactive protein was first described in the early 1930s. This acute phase protein is released by the liver in response to inflammation or tissue insult and is widely used as a highly nonspecific marker of sepsis. In a study by Póvoa *et al*. performed in 112 ICU patients, a serum CRP >8.7 mg/dl showed a Se of 93% and Sp of 86% to detect the presence of infection. Adding a temperature >38.2°C to this threshold increased Sp to 100% [51].

The latter authors also observed that CRP concentrations increased over time in patients with infection, yet remained unchanged in non-infected patients. A daily CRP variation of at least 4.1 mg/dl was predictive of nosocomial infection with a Se of 92% and Sp of 71%; when combined with a serum CRP above 8.7 mg/dl, these values increased to 92 and 82%, respectively [52]. Similarly, in patients with CRP concentrations >10 mg/dl on ICU admission, a decrease in CRP after 48 h was linked to a mortality rate of 15%, while its increase was associated with a mortality rate of 61% (p < 0.05) [53].

The peptide procalcitonin is synthesized by monocytes that are in the process of adhesion. PCT levels rise when there is local or systemic bacterial infection but not in the presence of a virus or autoimmune disease. Thus, PCT is more specific than CRP for detecting bacterial infection.

In a recent prospective study, on day 1 after admission to a medical-surgical ICU, a cut-off PCT >1.39 ng/ml showed the best area under the curve (AUC) for diagnosing sepsis (87%) and levels were found to significantly drop from day 1 to day 2 in survivors [54]. In addition, high PCT levels have been linked to an increased risk of mortality. As an example, in a recent prospective multicentre observational study performed in 1156 Greek in-patients, a PCT > 0.85 ng/ml was associated with 45% mortality in ICU patients [55]. It would appear that as for CRP, trends in PCT observed over time are more useful than single measurements [56].

However, we have yet to find a marker specific enough to provide a true diagnosis of BSI. The Surviving Sepsis Campaign 2012 guidelines state that the utility of PCT levels or other biomarkers to differentiate acute inflammatory patterns of sepsis from other causes of generalized inflammation (e.g., postoperative, other forms of shock) remains to be demonstrated [57].

### Rapid diagnosis of ventilator-associated pneumonia

Hospital-acquired pneumonia (HAP), especially ventilator-associated pneumonia (VAP), is one of the leading causes of infection and death in the ICU [58]-[62]. The incorrect or delayed treatment of HAP within a few hours gives rise to a worse prognosis and a higher mortality rate [63]-[65]. Useless antibiotics are also a cause of adverse events and unnecessary expense [66]. Thus, the etiologic diagnosis of VAP is a microbiological emergency because of its impact on the morbidity and mortality of this disease.

Bacterial identification and AST take 2 or 4 days, so there is a need for rapid diagnostic procedures. Rapid information is clearly more beneficial to the patient than more complete but delayed information. Gram staining, quantifying microorganisms in polymorphonuclear cells in bronchoalveolar lavage samples, and antibiograms conducted directly on clinical samples may provide information that correlates with subsequent culture results.

New diagnostic techniques, such as real-time PCR assays and "in situ" hybridization of bacteria, have been developed to speed up the identification of the pathogens responsible for this disease [67],[68].

### Lower respiratory tract samples for microbiology

All patients suspected of having VAP should undergo lower respiratory tract (LRT) sampling followed by a microscopy examination and culture of the specimen [69]. Deciding upon the best type of sample for diagnosing VAP is controversial and at present no sampling procedure has proved meaningfully superior to the rest [70]-[72]. Culture samples should ideally be transferred to the Microbiology Department within 30 minutes of collection to avoid a delay in processing and bacterial overgrowth [73],[74]. Storing LRT specimens refrigerated or frozen for 24 hours is an acceptable alternative when culturing cannot be performed immediately [75]-[77]. Despite this possibility, we would warn against this practice since any delay in receiving information will have devastating clinical consequences.

### Laboratory processing of samples upon arrival. Gram stain

There is still much controversy over the value of the Gram stain for anticipating the microbiological diagnosis of VAP. The medical literature is replete with varying data on the sensitivity (57-95%), specificity (48-87%), positive predictive value (PPV) (47-78%), negative predictive value (NPV) (69-96%) and accuracy (60-88%) of the Gram stain in the management of patients with VAP [78]-[82].

Some authors claim that a negative endotracheal aspirate (EA) Gram stain is of great negative predictive value for the diagnosis of VAP and may guide the decision to not initiate or to limit antibiotic treatment until culture results become available [78],[80],[83],[84]. Our opinion is that immediate reporting to the responsible clinicians of the result of a Gram stain on LRT secretions obtained by tracheal aspiration may help guide early treatment. At our Microbiology Department, the diagnostic validity of the Gram technique on EA in patients with suspected VAP has been estimated at: sensitivity 91%, specificity 61%, PPV 50.5%, NPV 94%, test accuracy 70%, positive likelihood ratio (PLR) 2.3, negative likelihood ratio (NLR) 0.14, and a post-test probability of a negative result of 6% [85]. This means that a negative Gram stain makes it highly unlikely that a positive culture result will be obtained the next day.

As a complement to the Gram stain, quantifying the proportion of cells containing intracellular organisms has also been proposed as a rapid method for the diagnosis of VAP. A cut-off of >1-2% of "infected" cells in bronchoalveolar lavage (BAL) specimens rendered a sensitivity of 79-93.6% and a specificity of 82-100% [86]-[88]. Thus, the detection of intracellular organisms in BAL specimens can be described as a rapid specific test with a high positive predictive value, and is recommended by the British Society of Antimicrobial Chemotherapy to guide initial therapy (grade A recommendation) [89]. In addition, this test does not seem to be affected by antibiotic therapy given up to 72 hours prior to sampling [90]. Along these lines, the European care bundle for the management of VAP recommends immediate reporting of Gram stain findings in respiratory secretions, including "infected" cells [91].

The guidelines of the Society for Healthcare and Epidemiology of America (SHEA) and the Infectious Diseases Society of America (IDSA) published in 2008 recommend a Gram stain directly on the sample and the quantitative culture of an EA or a BAL sample [92].

### Are there any other rapid direct methods that provide useful information before culture results become available?

Although it is widely accepted that the prognosis for a patient with VAP depends on the antibiotic susceptibility of the causative pathogen and on the time elapsed since its diagnosis and the first dose of effective antibiotic received [65],[93], there is presently no rapid procedure other than those mentioned whose efficacy in the management of VAP has been reliably proven. In the specific field of VAP, there is a clear need to address new molecular techniques that can detect one or several microorganisms [94] or rapidly identify certain resistance mechanisms directly on clinical samples. We recently obtained excellent results for the rapid diagnosis of VAP due to methicillin-resistant or susceptible *Staphylococcus aureus* (MRSA, MSSA) by directly subjecting clinical samples to PCR (GeneXpert, Cepheid® Inc., Sunnyvale, CA) [95]. This simple procedure shows a high diagnostic efficiency and can shorten the time to adequate antibiotic treatment. These results have also been validated by other authors [96],[97]. However, the GeneXpert kit has not yet received CE mark approval for this purpose. The ideal VAP molecular diagnostic assay should target various microorganisms and resistance genes, including *S. aureus*, *Pseudomonas aeruginosa*, *Acinetobacter baumannii*, a DNA sequence common to all *Enterobacteriaceae*, and the resistance genes *mecA*, *bla*_KPC_, *bla*_IMP_, *bla*_VIM_ and *bla*_OXA_ [98].

### Rapid preliminary cultures and susceptibility testing (VAP E-test)

Conventional processing of a secretion sample for microbiological investigation usually takes from 2 to 4 days. After inoculation and incubation for 24-48 hours, bacterial counts are performed and strains are isolated for pure culture. This is followed by pathogen identification and AST, which delays the results at least a further 24 hours. To this process, we would need to add the time of delays in transmitting information and in making therapeutic decisions.

In a study conducted at the Hospital Gregorio Marañón (Madrid, Spain), we compared the results of a direct E-test antibiogram for 6 antibiotic agents conducted on clinical LRT samples to those obtained by the standard AST. The E-test antimicrobial susceptibility procedure is a quantitative method for AST that consists of a plastic strip with a predefined gradient of antibiotic. The stable gradient provides inoculum tolerance where a 100-fold variation in cfu/mL has minimal effect on the minimum inhibitory concentration (MIC) of susceptible strains, and allows its application directly to clinical specimens [99]. The six antibiotics we used were oxacilin, cefepime, imipenem, piperacillin-tazobactam, amikacin and ciprofloxacin. Susceptibility data, obtained in 18 to 24 hours, were found to concur with those of the standard procedure at 48 to 72 hours in 98% of cases [100]. In a subsequent study, we confirmed the more effective and reduced use of antibiotics in VAP patients associated with the use of this quick procedure [101].

A new approach recently developed at our hospital is a modification of the direct E-test technique using a prototype chromogenic agar medium (Mueller-Hinton base) to generate both rapid antibiotic susceptibility and organism identification results [102],[103]. In a preliminary investigation of 143 LRT samples, 92.7% of the isolates were rapidly identified in this medium after 18 hours and 100% after 24 hours of incubation. Full agreement with the standard procedure was observed in 94.9% (Cercenado *et al*., unpublished data). Although these data are preliminary, we consider the use of chromogenic agar medium for E-tests on LRT samples is an improvement over the use of conventional Mueller-Hinton agar.

### Other diagnostic markers of VAP

The use of biomarkers such as CRP to more objectively and specifically diagnose VAP has also been assessed. Lisboa *et al*. used CRP as a diagnostic and prognostic marker, as well as to assess antibiotic treatment appropriateness [104]. These authors noted that the CRP coefficient (defined as the ratio between CRP levels on follow-up and CRP levels at baseline) decreased in patients receiving adequate treatment and that a coefficient of 0.8 at 96 hours post treatment onset was a good indicator of the appropriateness of antibiotic treatment (Se 77%, Sp 87%, area under the ROC curve 86%, 95% CI 75-96%). Unfortunately, CRP is a nonspecific biomarker of inflammation and may also be elevated in the presence of pulmonary infiltrates of non-infectious cause [74].

Regarding PCT, it is not a good marker for the diagnosis of VAP [105]. However, in VAP, this marker has been described as prognostic with elevated levels indicating a more severe clinical course and sustained high levels during the first week of illness indicating a worse outcome [106]. Some studies have also correlated a drop in PCT with a favorable outcome [107],[108] and reduced antibiotic consumption [109], although in other studies, neither PCT threshold values nor their kinetics were able to predict VAP survival [110],[111].

Despite these discrepancies, PCT seems to be a good indicator of bacterial load in patients with VAP. Most importantly, a low level of PCT is thought to accurately reflect controlled bacterial infection [74].

Other proposed biomarkers are the soluble triggering receptor expressed on myeloid cells-1 (Strem-1) [112] and interleukin-1beta and interleukin-8 in BAL fluid [113]. Chastre *et al*. recommend that PCT and Strem-1 should only be used to complement standard microbiological diagnostic tests. However, knowledge of serum PCT and Strem-1 levels may prompt a change in treatment early in the course of VAP and such findings have been used to step-up treatment when levels remain high or to avoid long courses of antibiotics when the levels of these markers rapidly fall [114]. Whether PCT and/or Strem-1 guidance can reduce antibiotic use in such a setting is yet to be seen, but the strategy appears promising [112],[115].

### The rapid diagnosis of urinary tract infection

The turnaround time for microbiological confirmation of a urinary tract infection (UTI) in a urine culture is not usually as critical as in life-threatening diseases like sepsis. Still, microbiological confirmation of a UTI takes 24-48 hours. In the meantime, patients are usually given empirical antibiotics, sometimes inappropriately.

### Rapid UTI screening methods. The Gram stain

The usefulness of Gram staining of fresh uncentrifuged urine to detect significant bacteriuria was first demonstrated in 1968 [116], and it has since been used as a screening test for UTI [117]-[119]. The accuracy of Gram staining for the diagnosis of UTI has been reported in the literature as: sensitivity 82.2-97.9%; specificity 66.0-95.0%; PPV 31.6-94.3%, and NPV 95.2-99.5%, varying with the different counts of microorganisms in the sample [118]-[122]. As with other rapid screening tests, accuracy is higher for greater bacterial counts.

The benefits of direct Gram staining of urine samples sent for culture are clear: it shortens the turnaround time for reporting negative culture results and guides empirical antibiotic treatment when microorganisms are seen. In addition, when compared to alternative rapid screening tests, the Gram stain has a higher accuracy [119],[123] and lower cost [122].

The use of the Gram stain has not been generalized because it needs more equipment and time than dipstick analysis, and is unlikely to replace dipstick testing across all health-care settings [123]. Skilled laboratory personnel are needed to correctly evaluate smears [124]. Yet, in laboratories where stained smears are part of the routine microbiological examination of urine samples, the time necessary to perform the stain and examine the slide under the microscope is relatively short [124].

MALDI-TOF mass spectrometry has been successfully used to rapidly identify culture-isolated microorganisms [42],[43] but has been little used directly on clinical samples except positive blood cultures and urine samples [48],[125]-[128].

We recently assessed the capacity of subjecting urine samples to sequential Gram staining and MALDI-TOF MS to anticipate clinically useful information [129]. From May through June 2012, 1,000 random urine samples from patients with a suspected UTI were Gram stained, and those returning bacteria of a single morphotype were subjected to MALDI-TOF MS. This procedure was correlated with standard semiquantitative urine culture results and the outcomes recorded as: match (information anticipative of culture result), minor error (information partially anticipative of culture result), or major error (information incorrect and potentially leading to inappropriate antibiotic therapy decisions). Results were available in 1 hour. Information anticipative of culture results was provided in 83% of cases, information with minor errors in 13% and information with major errors in 4%. For 96% of urine samples from patients with suspected UTI, the sequential procedure provided information that was consistent or showed minor errors. In future work, the clinical impacts of this rapid UTI diagnosis strategy need to be assessed in terms of factors such as a reduced time to appropriate empirical treatment or earlier withdrawal of unnecessary antibiotics.

### Anticipation of antibiotic susceptibility with direct testing

The practice of performing direct AST of urine specimens has the advantage of next-day reporting of antimicrobial susceptibilities. Direct AST of urine samples has proved to be as effective as standard methods, providing results 24 hours in advance with similar costs [130]. However, this method is criticized because the inoculum is not standardized and because sometimes a mixture of microorganisms can be found in the sample. Nevertheless, it has been used for many years with excellent results [131]-[136] and correlates well with reference methods. The fact that this method can confirm the appropriate antibiotic treatment in only 24 hours translates to the reduced use of wide-spectrum antibiotics with the consequence of diminishing antibiotic resistance.

### Rapid diagnosis of skin and soft tissue infections

According to the IDSA 2013 guide to the diagnosis of infectious diseases [137], cultures are not indicated for uncomplicated common forms of skin and soft tissue infections (SSTIs) (e.g., cellulitis, subcutaneous abscesses) treated in the outpatient setting. Whether cultures are beneficial for managing cellulitis in the hospitalized patient is uncertain and the sensitivity of blood cultures in this setting is low. Cultures are however recommended for the patient who requires operative incision and drainage because of the risk of deep structure and underlying tissue involvement [138]. The IDSA guide includes recommendations for sampling and processing specimens for a microbiological diagnosis of the most frequent SSTIs. Basically, the quality of the sample and the number of potential pathogens to be considered is first established in a Gram stain, and this is followed by a conventional culture procedure. These still traditional procedures are not rapid.

The recent availability of a rapid-detection assay to identify MRSA from wound specimens allows for better-informed therapeutic decisions. The Xpert MRSA/SA skin and soft tissue infection assay (GeneXpert, Cepheid® Inc., Sunnyvale, CA) is approved for rapid detection (within 1 h) of MRSA and MSSA in wounds. In a multi-center evaluation that included a total of 114 wound specimens, the MRSA/SA SSTI assay showed a Se of 97%, a Sp of 96%, a PPV of 92% and a NPV of 99% for MRSA detection; similar percentages were noted for MSSA [139]. Overall agreement between the assay and standard culture was 96.5%.

The GeneXpert kit directly applied to synovial fluid and tissue specimens (e.g., bone, muscle, fascia, etc.) has also proved useful for the diagnosis of osteoarticular and chronic prosthetic joint infections due to staphylococci [140],[141], though it has not yet received CE mark approval for this purpose.

The rapid identification and differentiation of MRSA in a wound specimen allows clinicians to more rapidly initiate appropriate antimicrobial therapy.

The steps recommended for the early diagnosis of a SSTI by *Streptococcus pyogenes* are: direct Gram staining of skin biopsies, tissues, fascia, muscle, purulent exudate or joint aspirates and the rapid detection of capsule and protein antigens in skin and/or tissues using available kits that show a Se of 60% to 91% and a Sp of 85% to 98% [142]-[144].

### Other rapid microbiological tests that may provide useful information in ICU patients

Besides the tests already mentioned, other diagnostic tests used in Microbiology can expedite the diagnosis of infection in these patients.

Those most often used in clinical practice, which also show adequate diagnostic performance, are the detection of the antigens of *Streptococcus pneumoniae* [145] and *Legionella pneumophila* serogroup 1 in urine [146] for patients with pneumonia; the detection of some viruses such as influenza and other respiratory viruses, or enterovirus and other central nervous system viruses [147]; and the detection of *Mycobacterium tuberculosis*, which in some cases is accompanied by the identification of resistance genes [148]. The detection of respiratory viral agents includes single or multiple pathogens (multiplex panels), which is highly convenient since most of these agents cause similar symptoms.

## Conclusions

Much progress has been recently made in the rapid etiologic diagnosis of infectious diseases. Some of the new approaches available are even able to detect antimicrobial resistances and this allows for treatment optimization, especially in the most vulnerable patients such as those admitted to the ICU. Current microbiology has shortened turnaround times in the treatment of many types of infection, such as sepsis, pneumonia, urinary tract infections, skin and soft tissue infections, viral infections or tuberculosis. Molecular techniques still have issues that need to be dealt with such as their limits of detection and sensitivity for certain samples and certain situations, their correlation with adequate diagnostic gold standards, their clinical validation and the correct interpretation of results, and the risk of contamination. Improvements are also needed in terms of widening the spectrum of pathogens and resistance mechanisms that may be identified or the sample types these procedures can be used on. Despite these limitations, the future of the field of molecular techniques for the rapid diagnosis of infections is highly promising.

## Authors' contributions

EB and AB took primary responsibility for the literature search, drafted the manuscript, wrote the manuscript, critically revised the manuscript, read and approved the final version.

## Abbreviations

AST: Antibiotic susceptibility testing
AUC: Area under the curve
BAL: Bronchoalveolar lavage
BCB: Blood culture bottles
BSI: Bloodstream infection
CA-UTI: Catheter associated urinary tract infection
CE: Conformité Européenne, meaning "European Conformity". It is a mandatory conformity marking for certain products sold within the European Economic Area (EEA) since 1985
cfu/mL: colony forming units/millilitre
CR: Catheter-related
CR-BSI: Intravascular catheter-related bloodstream infection
CRP: C-reactive protein
CVC: Central venous catheter
DTTP: Differential time to positivity
EA: Endotracheal aspirate
HAP: Hospital acquired pneumonia
ICU: Intensive care unit
LRT: Lower respiratory tract
MRSA: Methicillin-resistant *Staphylococcus aureus*
MSSA: Methicillin-susceptible *Staphylococcus aureus*
NAT: Nucleic acid testing
NLR: Negative likelihood ratio
NPV: Negative predictive value
PBS: Phosphate-buffered saline
PCR: Polymerase chain reaction
PCT: Procalcitonin
PLR: Positive likelihood ratio
PPV: Positive predictive value
ROC curve: Receiver operating characteristic curve
Se: Sensitivity
Sp: Specificity
SSTI: Skin and soft tissue infection
sTREM-1: Soluble triggering receptor expressed on myeloid cells-1
UTI: Urinary tract infection
VAP: Ventilator associated pneumonia
